# Totally Extraperitoneal Approach With Preperitoneal Repair for the Treatment of Midline Hernia Defects: A Case Series and Single-Center Experience

**DOI:** 10.3389/jaws.2025.14611

**Published:** 2025-06-09

**Authors:** Aritz Equisoain-Azcona, Javier García-Quijada García, Ramón Sanz-Ongil, Carlos Bustamante-Recuenco, Lucía Martínez-Minuesa, Álvaro Sobrino-Grande, Agustín Bertomeu-García, Francisco Javier Angulo-Morales

**Affiliations:** ^1^ Universidad Alfonso X el Sabio, Hospital Central de la Cruz Roja, San José y Santa Adela, Madrid, Spain; ^2^ Hospital Universitario de Getafe, Getafe, Spain; ^3^ Hospital Central de la Cruz Roja, San José y Santa Adela, Madrid, Spain

**Keywords:** PeTEP, midline hernia, rectus diastasis, morbidity, recurrence rate

## Abstract

**Introduction:**

The introduction of laparoendoscopic techniques in abdominal wall surgery has transformed this field, growing exponentially in the last decade. The totally endoscopic preperitoneal approach (PeTEP) may offer advantages over traditional techniques by allowing complete anatomical restoration with lower morbidity. In order to evaluate the efficacy and safety of this novel technique, we reviewed our results 1 year after its implementation.

**Material and methods:**

We perform a retrospective observational study including all patients aged over 18 years with midline hernias <8 cm with or without associated rectus diastasis who underwent PeTEP repair between March and December 2024 in our center. The evaluated outcomes included demographic characteristics, operative time, mesh size, length of hospital stay and morbimortality and recurrence rate.

**Results:**

Seventeen patients (10♂, 7♀) were included, with a mean age of 49.82 years (SD: 12.43). Multiple defects were observed in 88.2% of cases, with the M2-M3 combination being the most frequent (41.2%). The mean defect diameter was 2.88 cm (±1.62). The superior access was the most frequent (66.7%), and a mesh with a mean height of 29.71 cm and a mean width of 14.41 cm was used. No conversions to open surgery were recorded, although three cases (17.6%) required a change to the retromuscular plane (eTEP-RS), being all of them female patients. Complications were limited to two superficial hematomas (11.8%). The median hospital stay was 1 day. With a median follow-up of 87 days (IQR 143.5), no recurrences were detected.

**Conclusion:**

Our results suggest that the PeTEP approach is a safe and effective technique for small to medium-sized hernias with or without rectus diastasis. Additional studies with long-term follow-up and comparisons with pre-existing techniques are needed to confirm its benefits and establish its indications.

## Introduction

Ventral hernias represent a significant health issue, not only due to their high incidence but also because of the inherent complications associated with them and those arising from their surgical repair [1]. The introduction of endoscopic techniques in Abdominal Wall surgery has been revolutionary, experiencing exponential growth over the past decade [2]. Minimally invasive approaches have minimized problems associated with open repair, such as a higher incidence of surgical site infection, postoperative pain, and prolonged recovery times, by offering advantages such as smaller incisions, reduced pain and faster recovery [3].

However, conventional laparoscopic techniques, such as intraperitoneal mesh repair (IPOM, IPOM+; LIRA), although beneficial, present associated risks such as adhesion formation and mesh erosion [3]. This has led to the development of alternative approaches that allow for the placement of large mesh while achieving midline closure and isolating the prosthesis from abdominal cavity: transabdominal preperitoneal (TAPP) [4] and enhanced-view totally extraperitoneal Rives Stoppa (eTEP-RS) [5]. Recents studies have demonstrated the potential of endoscopic extraperitoneal repair in reducing complications such as postoperative pain compared to intraperitoneal techniques without significant differences at any other level (seroma, hematoma, infection, recurrence, or readmissions) [6–8].

One of the most recently described techniques is the preperitoneal extended-view totally extraperitoneal (PeTEP) [9]. This approach preserves the integrity of the muscular complex, as it maintains the medial and lateral insertions of the posterior rectus sheath, which enhance the postoperative functionality of the abdominal wall [10, 11]. Those improvements could overcome functionally consequences of the previous eTEP-RS technique [12, 13]. Furthermore, positioning the mesh behind the posterior rectus sheath distances it from neurovascular bundles and epigastric vessels, potentially reducing complications such as bleeding and postoperative pain. Finally, this technique allows for a totally preperitoneal repair with an overlap equal to or greater than that achieved with the eTEP-RS technique or the conventional Rives-Stoppa, combining the benefits of endoscopic techniques with midline closure [9].

Despite the potential advantages described above, only three PeTEP case series have been published to date [9, 11, 14], and it has been primarily described for primary midline defects associated with rectus diastasis. Our objective is to evaluate the efficacy and safety of PeTEP in the treatment of primary or incisional hernia defects by analyzing the technical details and the outcomes related to morbimortality and recurrence rate.

## Material and Methods

### Study Design and Patient Selection

We performed a retrospective observational study. All patients who underwent PeTEP surgery from March 2024 to December 2024 were included. We registered demographic (age, gender, BMI, ASA score, arterial hypertension, diabetes mellitus, hyperlipidemia, cardiac or pulmonary disease and previous abdominal surgery), hernia characteristics based on the European Hernia Society (EHS) [15], mesh type and size, operative technique, conversion rate, as well as postoperative data: overall complication rate, type of complications based on the Clavien Dindo classification [16], SSI, SSO, hospital stay, recurrence rate and follow-up time. All surgical procedures were completed by two surgeons in a single institution.

The inclusion criteria encompassed adult patients presenting with solitary primary or incisional ventral hernias, with a maximum defect diameter ranging from 3 to 8 cm, as well as individuals with multiple midline defects or any defect accompanied by symptomatic rectus diastasis. All patients eligible for surgery underwent a CT abdomen scan as part of the preoperatory assessment. The measurement of the hernia defect and the rectus diastasis was performed with the CT scan and the surgical description. In case of multiple defects, the width reported was the hernia complex or the largest defect.

Approval of the local investigation and ethics committee was obtained prior to the beginning of the study. We followed the recommendations of the STROBE guidelines [17] during the project development.

### Surgical Technique

The procedures were performed under general anesthesia with orotracheal intubation. Preoperative antibiotic prophylaxis with cefazolin was administered according to the standard protocol of our center. Thromboprophylaxis was also provided using compression stockings and postoperative low-molecular-weight heparin (LMWH), following the recommendations of the Caprini risk assessment scale.

We distinguished two types of access for the treatment of midline defects. Decision was made based on the patient’s characteristics and the pathology being addressed.

### Superior or Infraxiphoid Access

#### Patient Positioning

We follow the steps outlined by Munoz and collaborators for superior access [14]. Thus, the surgeon and the assistant are positioned at the patient’s head, with the monitor screen placed at the foot. The patient is placed in the supine position with legs adducted and arms extended alongside the body.

**FIGURE 1:**
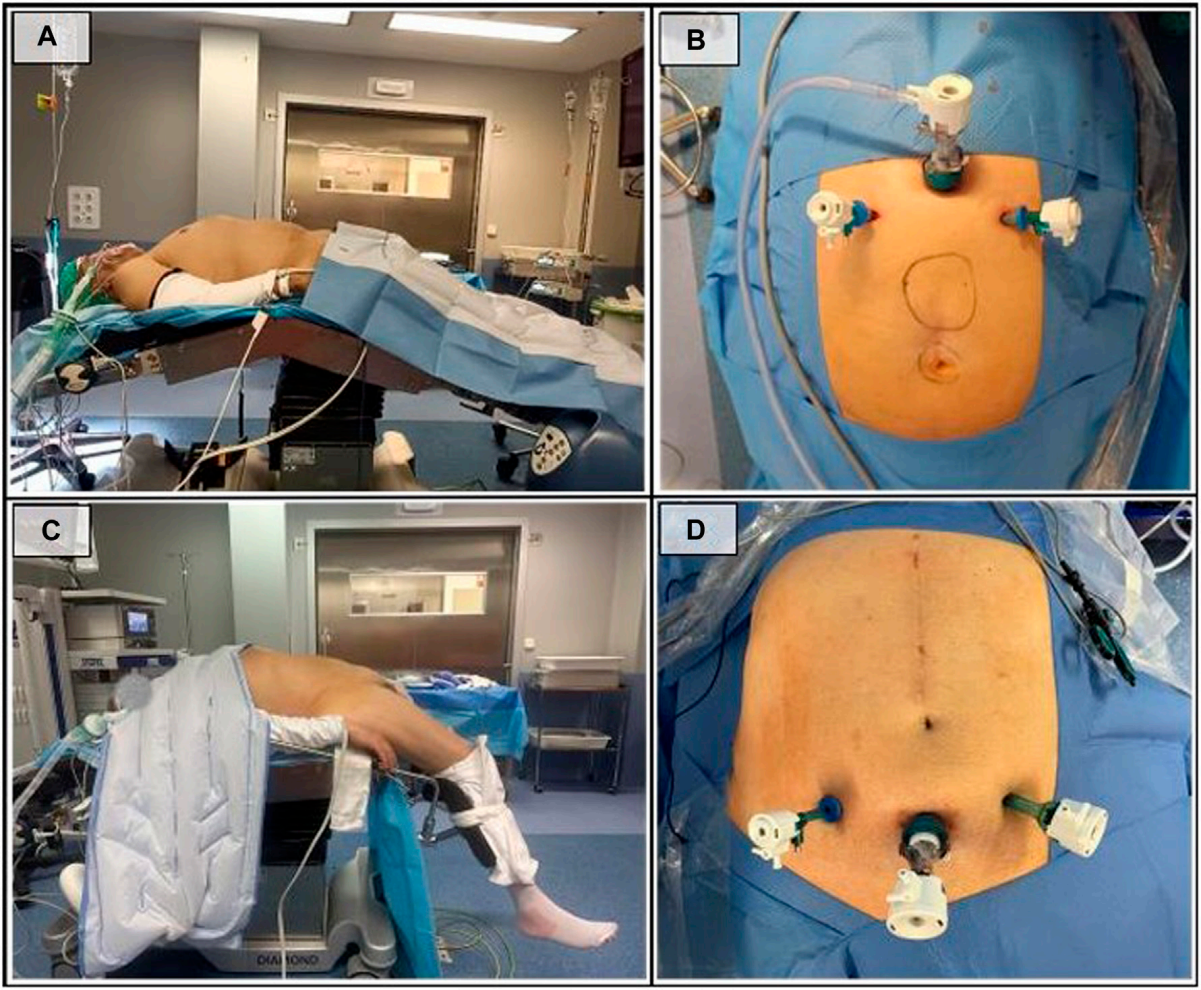
Patient and trocars placement. **(A)** Patient position in the superior access; **(B)** Trocar placement in the superior access; **(C)** Patient position in the inferior access; **(D)** Trocar placement in the inferior access.

Moderate lumbar hyperextension is then applied using the surgical table (Figure 1A). This position is essential to increase the motion range of the dissection instruments and to improve ergonomics.

#### Dissection Phase

As in the eTEP-RS technique, dissection is performed centripetally, initially creating the working space and subsequently dissecting the hernia defect.

First, an incision is made 1 cm below the xiphoid process to expose and open the linea alba, providing access to the preperitoneal subxiphoid fatty rhomboid. A gentle digital dissection is then performed to create the initial preperitoneal space, followed by the insertion of a 12 mm Hasson trocar. Subsequently, the dissection is extended under direct vision using the optical device, allowing for the placement of two 5 mm trocars in the hypochondria (Figure 1B). Insuflation pressure is fixed at 10–12 mmHg, with a low CO2 flow (3 mmHg) at the beginning of the surgery. These parameters are modified only if necessary.

**FIGURE 2:**
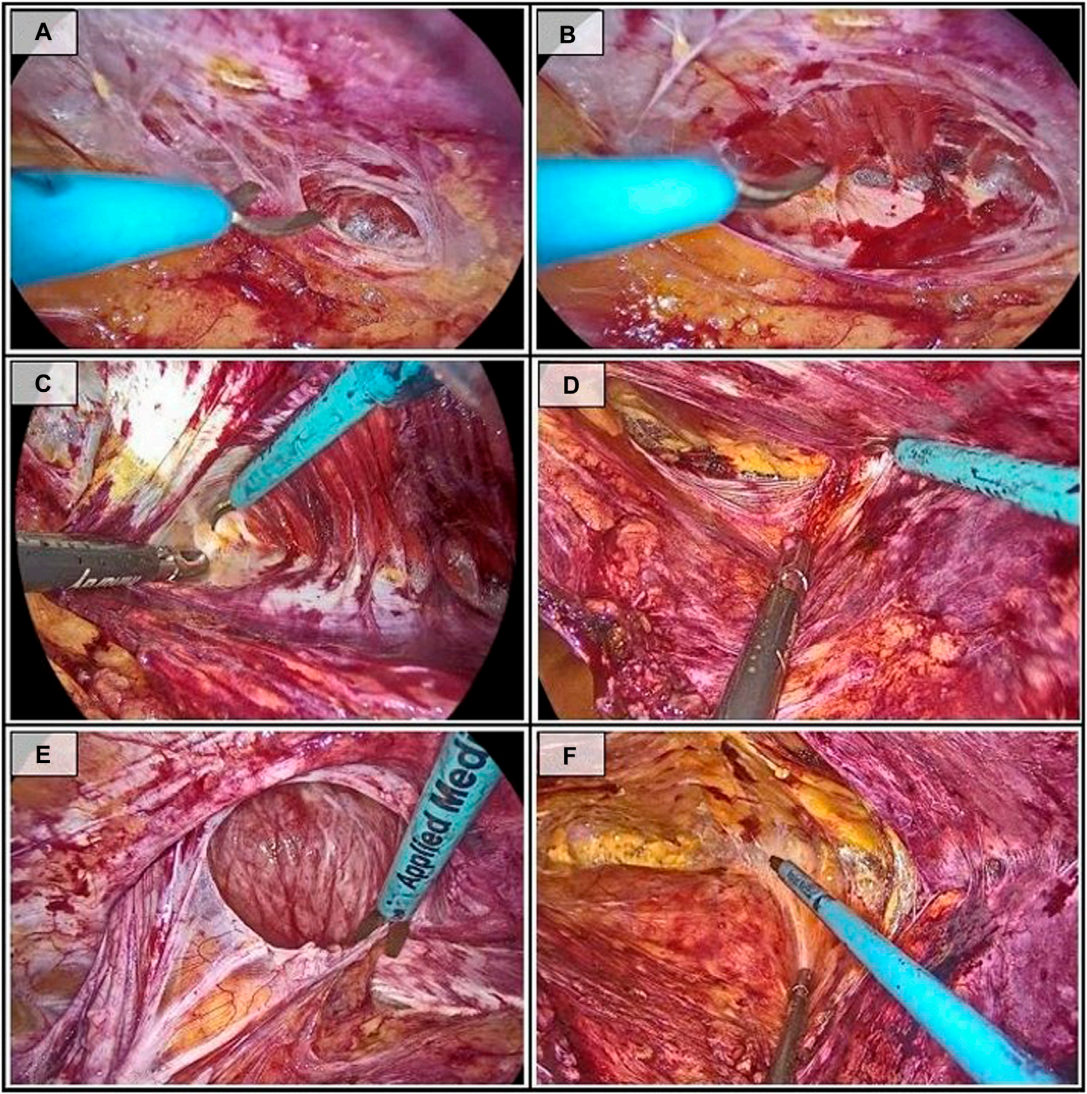
Sequential steps during the dissection in the superior access (right side). **(A)** Sectioning of the transversalis fascia; **(B)** Exposure of the transverse muscle; **(C)** Pretransversalis dissection following the insertion of the transverse muscle; **(D)** Pretransversalis dissection completed at the arcuate line; **(E)** Reduction of the hernia sac; **(F)** End of the dissection in the retropubic preperitoneal space.

The next step involves lateral access to the pretransversalis space, sectioning the transversalis fascia and exposing the transverse abdominal muscle (Figures 2A,B). The anatomical arrangement of the transverse muscle, whose insertion medializes in the most cranial portion, facilitates the localization of the muscle fibers and access to the pretransversalis space in this area.

The dissection continues in a laterocaudal direction, using the tendinous insertion of the transverse muscle as a reference point (Figure 2C), and progresses until reaching the arcuate line (Figure 2D).

Peritoneal tears are easy to cause at this stage. The key to preventing them is to first access the pretransversalis space laterally, where the fascia is thicker and there is more preperitoneal fat, and then progress towards the midline. In this way, in the event of a peritoneal opening, the dissection cavity is larger and therefore less likely to collapse. Additionally, maintaining the CO2 flow at a low intensity [3–5] also helps prevent this unfortunate event.

At the same time, the preperitoneal space of the midline is dissected. At this point, in addition to reducing the intra-abdominal contents, attempts will be made to reduce the hernia sac and the preperitoneal fat (Figure 2E). It is usually simple in primary defects whereas in case of incisional hernia the accidental rupture of the sac is relatively common. If this occurs, the peritoneal tear is extended to inspect the herniated content and safely reduce it.

In many cases, complete reduction of the sac is impossible due to fibrosis of the hernia defect so the entire peritoneal sac is sectioned and left in the subcutaneous tissue. Posteriorly, this abandoned sac will be incorporated into the suturing of the linea alba in a Venetian blind technique, thereby reducing the risk of postoperative seroma.

Subsequently, the pretransversalis space will be connected to the preperitoneal space, alternating blunt and sharp dissection in a latero-medial direction to minimize peritoneal tears.

Finally, dissection below the arcuate line of the preperitoneal space will be performed, preserving the inferior epigastric vessels laterally (Figure 2F). The dissection concludes with visualization of the pubic symphysis.

### Inferior or Suprapubic Access

#### Patient Positioning

In this access, the patient is positioned with low stirrups and hyperextension at the hip level (Figure 1C). The surgeon will be positioned between the patient’s legs, with the monitor screen at the head.

#### Dissection Phase

The procedure begins with a suprapubic open approach, performing blunt dissection of the retropubic preperitoneal space followed by the insertion of two accessory trocars in both iliac fossae (Figure 1D). Correct positioning of the trocars is crucial, as a trocar placed too low may significantly limit the ergonomics of the procedure and a trocar placed too high, near the arcuate line, makes initial access and pretransversalis dissection more difficult. Positioning it 2–3 cm above the pubis, or at the junction of the upper two-thirds and the lower third of the distance between the navel and the pubis, serves as a good reference to avoid these difficulties.

**FIGURE 3:**
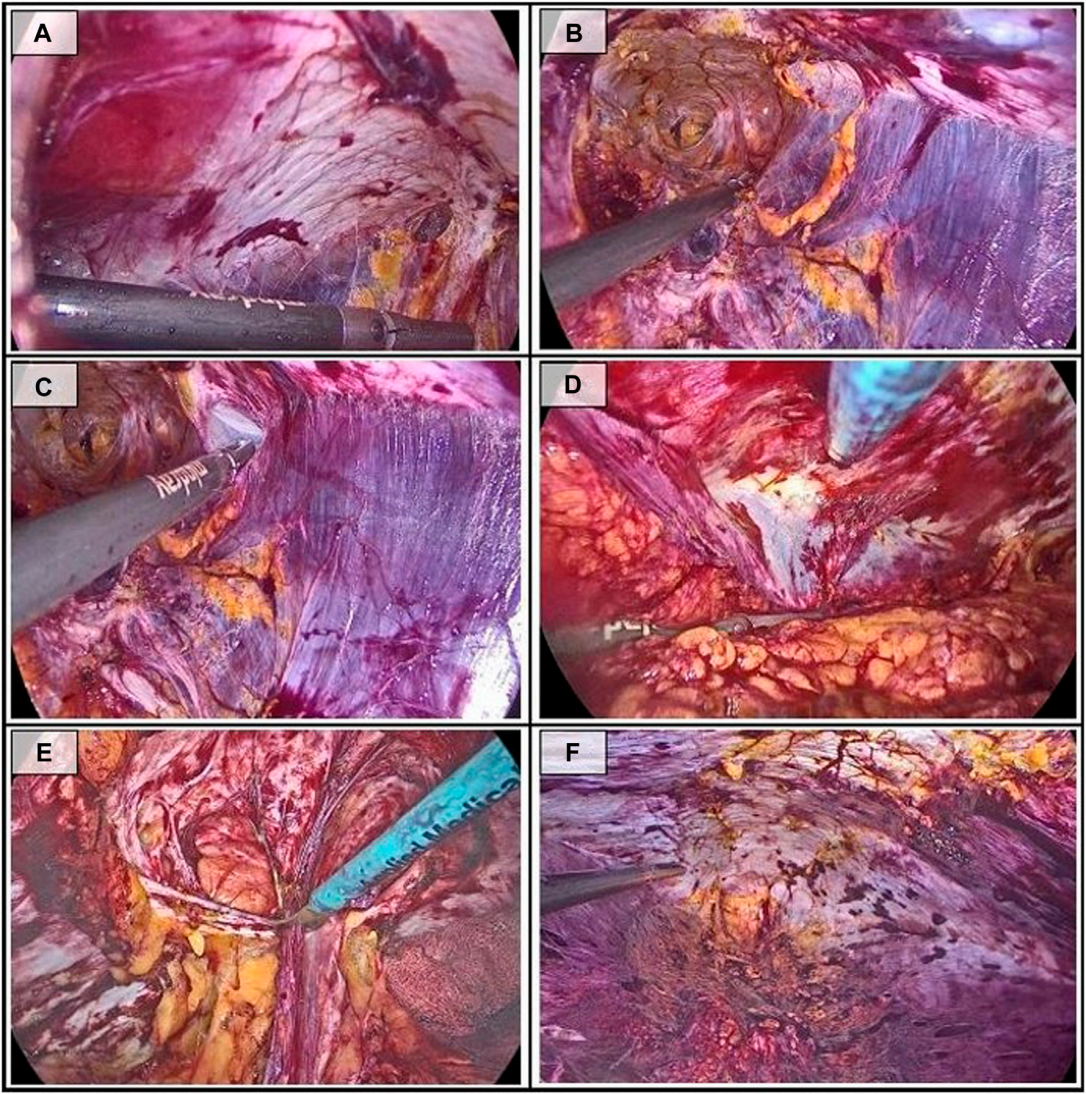
Sequential steps during the dissection in the inferior access. **(A)** Identification of the arcuate line; **(B)** Preperitoneal dissection of the linea alba; **(C)** Access to the pretransversalis space on the left side; **(D)** Pretransversalis dissection on the right side; **(E)** Reduction of the hernia sac; **(F)** Final view of the dissection.

At the start of the procedure, it is key to identify the arcuate line (Figure 3A). Accessing along the rectus muscles may incorrectly lead us to the retrorectal space, used in eTEP-RS.

The introduction of an 11 mm trocar in the left iliac fossa allows for changing the optics and facilitates dissection and suturing from the suprapubic trocar. This provides better ergonomics as it does not interfere with the patient’s thighs.

The dissection of the preperitoneal space underlying the linea alba does not differ from the approach described for the superior access (Figure 3B), although access to the lateral pretransversalis space (Figures 3C,D) may be more complex, with a higher risk of peritoneal tears. In this case, we recommend approaching it at the periumbilical level, where the transversalis fascia is more prominent and easier to identify.

As in the superior access, all hernia sacs and the accompanying preperitoneal fat are reduced during the midline dissection (Figure 3E). The dissection concludes upon reaching the preperitoneal subxiphoid rhomboid (Figure 3F).

### Common Procedure: Plication of Linea Alba and Mesh Placement

**FIGURE 4:**
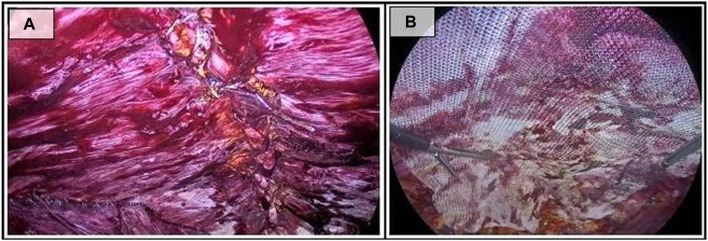
Common procedure steps. **(A)** Plication for reconstruction of the linea alba; **(B)** Placement of the mesh.

In both accesses, a repair of the midline will be performed by closing the hernia defects with slow absorbable barbed sutures, USP 0 or USP 1 (Figure 4A). During the closure of the hernia defect, it is recommended to include the peritoneal sac in case of partial reduction or the pseudosac otherwise, which will be lodged in the subcutaneous tissue in order to reduce dead space and minimize seroma formation. In cases where the patient presents rectus diastasis, a rectus plication with a barbed inverted suture is performed.

Once the integrity of the midline is restored, pneumoperitoneum pressure is reduced to 5–7 mmHg and closure of any peritoneal defects that occurred during dissection is performed. Due to the reduction in peritoneal tension after the aponeurotic repair, closing these defects is technically easier at this stage of the procedure.

Finally, the reinforcing mesh is placed. In our case, we use a medium weight polypropylene mesh approximately 30 cm long by 15 cm wide, without fixation. The mesh is placed against the muscular wall to avoid folds, and once extended, the progressive deflation of the cavity is carried out, ensuring proper placement (Figure 4B).

### Statistical Analysis

Satistical analysis was performed using SPSS 27.0^®^ software (IBM, SPSS Statistics for Windows, Version 27.0. Armonk, NY: IBM Corp.). Categorical variables were described as numbers and percentages. Due to the sample size, Shapiro–Wilk test was used to determine the Gaussian distribution. Continuous variables were presented as means and standard deviation if they followed a normal distribution or as median and interquartile ranges otherwise.

## Results

**FIGURE 5:**
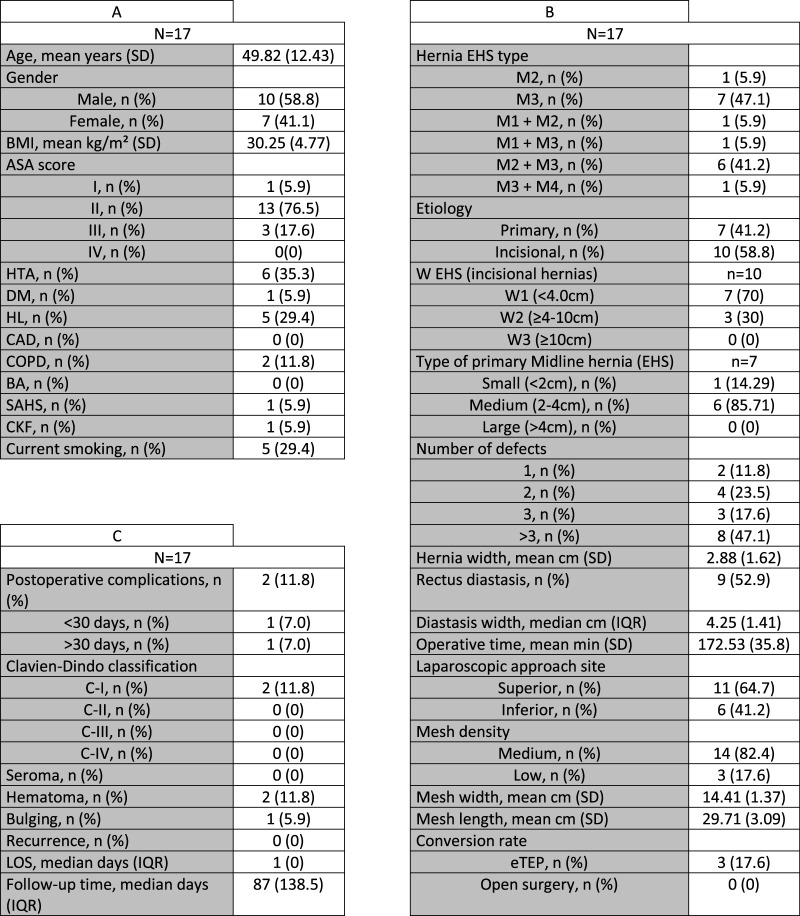
**(A)** Demographic data: BMI, body mass index; ASA, American Society of Anesthesiogists; HTA, Arterial Hypertension; DM, Diabetes Mellitus; HL, Hyperlipidemia; CAD, Coronary artery disease; COPD, Chronic obstructive pulmonary disease; BA, Bronchial asthma; SAHS, Sleep apnea-hypopnea syndrome; CKF, Chronic kidney failure; SD, Standard deviation. **(B)** Perioperative characteristics: EHS, European Hernia Society; eTEP, extended totally extraperitoneal; IQR, Interquartile range. **(C)** Postoperative course data: SSI, Surgical Site Infection; LOS, Length of stay.

A total of 17 patients were included (10♂, 7♀) with a mean age of 49.82 years (SD:12.43). ASA II score was the most frequent (76.5%), being the arterial hypertension (n = 6, 35.3%) and the hyperlipemia (n = 5, 29.4%) the most frequent comorbidities. A mean BMI of 30.25 (SD: 4.77) was registered, with 47.1% (n = 8) having a BMI≥30. Detailed demographic characteristics are shown in Figure 5A.

As shown in Figure 5B, and according to the EHS Hernia classification, epigastric and umbilical defects (M2 and M3) were the most frequent, with just two patients with a M1 defect (11.8%). Umbilical hernias coexisted with rectus diastasis in nearly half of the cases (n = 9, 52.9%). 15 cases (88.2%) had more than one wall defect. A mean hernia and rectus diastasis size of 2.88 cm and 4.25 cm was observed, respectively. In case of multiple defects or coexistence with rectus diastasis the medium diameter was 2.44 cm, whereas the two patients with single hernias had a diameter of 5.5 and 7 cm respectively*.* The most frequent indication for surgery was the incisional hernia (n = 10), with the remainder (n = 7) being primary hernias.

The infraxiphoid access was the preferred one in our series (64.7%). The mean operative time was 172.53 min, and a trend towards a longer surgery in patients with rectus diastasis was found (160.88 min vs. 182.89 min, p = 0.216). We used a medium density permanent mesh in nearly all the sample, with a mean width and length of 14 and 29 cm (range: 12 cm width and 38 cm length). Glue was used as fixation method just in one case, none was applied in the remainder. In 3 cases (17.6%) it was necessary to convert the technique to eTEP-RS to complete the surgery due to the fragility of the transversalis fascia. All of them were female patients. Conversion to open surgery was not necessary up to now. No postoperative drains were used in any of the patients.

At a median follow up of 87 days, postoperative course (Figure 5C) was uneventful in 15 of the 17 cases included. Only 2 hematomas were detected, and none of them required specific treatment (Clavien-Dindo I). Only three patients required a 2 day hospital stay due to pain management, being the median LOS 1 day (IQR = 0). There were no cases of internal or parietal hernias due to peritoneal rupture, and none required reintervention. No case of SSI, ileus, pulmonary or cardiac complications were detected. No recurrence has been observed in the study period.

## Discussion

Minimally invasive approach to abdominal hernia treatment has seen exponential growth over the past decade. The eTEP-RS technique has proven to be safe, effective, and reproducible in the treatment of abdominal wall hernias [5]. Despite the advantages offered by this technique, it presents some drawbacks, fact that has encouraged the development of preperitoneal techniques such as ventral TAPP or the novel PeTEP to address these issues.

According to comorbidity and anesthetic risk, our results are similar to those reported by Arias-Espinosa et al. [11], although it should be noted that there is a higher percentage of women (41.2% vs. 8%) in our series. Since adopting this technique in our center, we have observed that the transversalis fascia has less consistency in this subgroup, making its dissection more challenging and facilitating accidental ruptures of the peritoneum. This fact is reflected in the need for conversion to eTEP-RS in 3 of the 7 female patients operated on at our center. However, we believe that PeTEP is feasible in women, as the intervention could be completed in 57.15% of the cases, and in the remainder cases the conversion to eTEP-RS did not result in differences in morbimortality or hospital stay.

The original case series published by Valenzuela [9] includes only patients with primary hernias. However, his group did include 16% of incisional hernias (n = 4) when developing the robotic approach and Munoz-Rodríguez et al. reported a 37.5% in this field [11, 14]. This percentage is lower than observed in our series (58.8%, n = 10), although the average diameter of the parietal defect was similar to that reported by them (2.88 vs. 3 and 2.54 cm) [11, 14]. The absence of difference in terms of morbimortality and recurrence between incisional hernias and primary ones in the mentioned studies as well as in our series suggests that the etiology of the hernia may not be a limitation for this procedure.

Nevertheless, in our opinion, the predominant limitation of this technique is indeed the size of the defect. Its applicability for the repair of W3 defects may be constrained, as the absence of fascial release does not enhance the posterior sheath compliance and in these cases, BTA prehabilitation may not be enough to achieve the midline closure. However, this limitation is also present in ventral TAPP, which constitutes the other preperitoneal alternative. The difficulty in extending the dissection away from the peritoneal sheet section and the challenging ergonomics of this technique, in our opinion, represent similar constraints. Moreover, this approach is technically challenging, even more when performed without a robotic platform.

Regarding the surgical access, in our series the superior or subxiphoid approach was predominant (64.7%). The superior approach provides certain advantages in our opinion. On the one hand, the medialization of the semilunar line in the epigastrium and mesogastrium [18] allows visualization of the transversus muscle, fact that combined with the greater presence of preperitoneal fat at this level [19] facilitates access to the pre-transversalis space. Moreover, this access enhances ergonomics by preventing the instruments from interfering with the patient’s legs and allows for the repair of M4-5 hernias and concomitant inguinal hernias. On the other hand, M1 defects cannot be treated through this approach, and in certain cases the plication of the cranial edge of the linea alba is not possible. Also, subcostal trocar placement has a worse aesthetic result.

Since its first publication in 2017 [5], the eTEP-RS technique has revolutionized minimally invasive abdominal wall surgery by combining the less invasive nature of laparoscopic surgery with the advantages of extraperitoneal large mesh placement [1, 3, 7, 20]. Its efficacy and safety have made it the international benchmark in minimally invasive surgery and, therefore, the main point of comparison.

In the 2022 meta-analysis by Aliseda et al. [21], with a sample size of 918 patients, a similar rate of primary hernias (45.7% vs. 41.2%) to our series was observed, although the size of the aponeurotic defect exceeded ours by 3.5 cm (6.38 cm vs. 2.88 cm). We believe this difference is justified given that it is a developing surgical technique, although it should be noted that defects up to 7 cm were successfully repaired in our study. The conversion rate to open surgery ranges between 0% and 1% in the literature [21, 22]. In our series, this type of conversion was not necessary in any case, although in 3 patients the intervention was completed via eTEP-RS approach, as previously mentioned.

Regarding ventral TAPP, both the hernia dimensions (2.46–3 cm) and the conversion rate (0%) reported in the literature are more comparable to those of PeTEP [23, 24]. Its emergence as an alternative to IPOM in obese patients with small-to-medium defects may explain these similarity.

The data obtained regarding intraoperative complications (0%) and mean hospital stay (1 day) were similar to those reported in the literature [21, 22, 25]. It is worth noting the longer surgical time required for the PeTEP in our series compared to other published series (172 vs. 120 vs by Arias-Espinosa and 136 min by Munoz-Rodriguez) [11, 14] and compared to the standard eTEP-RS (148.89 min) and TAPP (90.2 min) [23]. This can be explained by the learning curve inherent to any new technique as well as the higher incisional hernia rate of our study.

Postoperative seroma is one of the most frequent complication in minimally invasive abdominal wall surgery. Some series have described up to 100% seroma in cases where active search was conducted [26]. In our series, no clinically evident seroma was detected. However, based on the aforementioned premise, it could be classified as a 0b seroma according to Morales-Conde’s classification [27], for it might be detected through radiological examinations.

Regarding postoperative complications, only two superficial hematomas (11.8%) were detected, and specific management was not required. This percentage is slightly higher than the 4%–5.8% referred to in the literature for PeTEP [9, 11, 14] [9,11], the 1%–3% for eTEP-RS [21, 22, 27] and the 5% for ventral TAPP [23]. The small sample size (n = 17) may justify these differences in our opinion. Regarding recurrences, as in the previously published series [9, 11, 14], we did not observe any during the study period, which represents an improvement compared to the 3.6% reported with ventral TAPP [28]. Nonetheless, the follow-up period did not exceed the 6 months in the PeTEP series mentioned. Thus, caution is demanded when interpreting this result.

In our series, no bulging was detected during the follow-up, result that contrast with the 17% rate referred in certain eTEP-RS studies [29]. In our judgement, this is explained by the preservation of the insertion of the posterior rectus sheath in the PeTEP, which allows for a complete conservation of the neurovascular pedicles and the musculoaponeurotic complex.

Our work has the limitations inherent to retrospective studies, including selection bias and the lower reporting of operative technical difficulties as the main ones. Additionally, being a single-center study with a short follow-up period limits its external validity and the reliability of the recurrence rate, respectively. In this regard, we aim to increase the sample size and follow-up time, as well as to conduct a comparative study against established techniques. Nonetheless, we believe its value as a pilot study is appropriate given the excellent results obtained regarding the proposed objectives and the detailed description of the surgical technique.

## Conclusion

Preperitoneal extended totally extraperitoneal (PeTEP) repair is a safe and effective technique in small to medium midline primary or incisional hernias, as shown by our low complication rate and the absence of recurrences. It is also a flexible approach, as a subxiphoid access is also possible and provides advantages in specific cases. However, longer follow-up is required to validate our results and further refine the indications for this technique.

## Data Availability

The raw data supporting the conclusions of this article will be made available by the authors, without undue reservation.
